# Dr. De Carro, on Vaccination

**Published:** 1804-08-01

**Authors:** J. De Carro

**Affiliations:** Vienna


*Letter from Dr. De Carro, to the Editors of the Bibliotheque Britannique.*

Gentlemen,

YOU have seen by my preceding letters, and my work on Eastern vaccination, what a rapid progress that happy discovery has made in Asia. Hitherto we have not heard that it was extended into the peninsula of Indostan; but a letter from Bombay informs me, that the vaccine practice is general from Cape Comorin to Delhi. I have just experienced a fresh gratification, that of having laid the foundation of the same practice in Persia. The occasion was as follows.

Dr. Milne, who is attached to the English factory at Bassora, after inoculating a great number of the children of that city, had experienced an interruption in procuring a supply of fresh matter, and had trusted to impregnated threads, lancets, and glasses. Having to his great mortification, found all these means fail, he wrote to me in the month of May last, earnestly requesting me to send him some matter by the earliest opportunity. With the utmost care, I collected it on ivory lancets, and impregnated lint with it, according to the excellent method of Messrs. Ballhora and Stromeyer. Meanwhile, Dr. Milne was obliged to quit Bassora, and to repair to Bashire or Abusheher in Persia. My packet, which was dispatched from Vienna at the beginning of August, not finding him at Bassora, was forwarded to him at Bashire, where it arrived about the end of November. Dr. Milne and Mr. Jukes, an English surgeon, who despaired of success with matter nearly four months old, were agreeably surprised to find the lint impregnated with a matter that was yet liquid, and which produced the desired effect the first time they made trial of it. The ivory lancets, on the contrary, produced no effect at all.

I know not, in the history of the cow-pox, any more satisfactory satisfactory examples of the benefit arising from care and attention in the manner of collecting the vaccine matter, than the success of the packets which I sent some time since to Bagdad and lately to Bashire. We have seen the incalculable benefit produced by the former; and I have every reason to hope, that the latter, which has been so happily transmitted to Persia, will produce effects equally salutary in that extensive and celebrated empire.

Dr. Milne and Mr. Jukes write me from Bashire, on the 11th and 15th of January, that their first success has made the most powerful sensation in that town, which now carries on the greatest trade of any in the empire; and that the people flock to them to have their children vaccinated.* They likewise inform me, that a mission is on the point of setting off for Tehran, the seat of the government, and that Mr. Jukes, who is to accompany it in the quality of surgeon, has taken the most effectual measures, to procure the patronage of the governors of provinces in favor of the cow-pox, and even to explain the history of its utility to the sovereign to whom they will be presented. I am extremely impatient to receive farther accounts of this expedition. Mr. Jukes has engaged to correspond directly with me. Dr. Milne, who has been called to Bombay, is succeeded at Bassora by Mr. Donald, who purposes doing all that lies in his power to promote the propagation of the cow-pox. The East India Company has taken into consideration my endeavours to introduce the vaccine practice into the British settlements; and the Secretary to the Court of Directors at London, has acquainted Mr. Paget, the Ambassador from England to the Court of Vienna, in a letter

* This is not yet the case in Europe, though so proud of her superiority in knowledge over the natives of the East. In a country where the government has bestowed the utmost attention to render vaccination general, the small-pox still continues to make dreadful havoc. The Canon Gattoni informs me, that in the small town of Como, his native place, notwithstanding the repeated invitations of the government, the exhortations of the clergy, the zeal and persuasions of Dr. Carloni, a physician appointed for the purpose of diffusing the vaccine practice, the intrigues of a few ignorant and envious professional men still prevail to such a degree, that in the two last months of 1803, nearly three hundred children, whose parents had obstinately refused to suffer them to be inoculated with the vaccine, were carried off by the small-pox. The government testified its grief on the occasion by a solemn proclamation. But as the distemper spared all the children who had been vaccinated, it is to be hoped that such a striking instance of the excellence of this preservative will not be lost, but will prevent the recurrence of similar disasters.

letter dated December 9, 1803, that the Directors have voted two hundred guineas to purchase a piece of plate for me.

Now I am speaking of presents, I have likewise received from the Hospodar of Moldavia a superb Indian shawl, which his serene highness accompanied with an extremely obliging letter that he was pleased to write me, and in which he gives me an account of the efficacious measures he has taken to propagate the cow-pox in that principality.

*Vienna*, I am, &c.

*March* 27, 1804. J. DE CARRO.

